# Spica magnetic resonance imaging for determination of abduction angle: initial results and reproducibility assessment 

**DOI:** 10.1007/s11832-016-0765-1

**Published:** 2016-08-12

**Authors:** M. J. Rivlin, J. H. Kan, E. K. Schallert, S. P. Jadhav, W. Zhang, S. B. Rosenfeld

**Affiliations:** 1 E.B. Singleton Department of Pediatric Radiology, Texas Children’s Hospital, Baylor College of Medicine, 6701 Fannin Street, Ste 470, Houston, TX 77030 USA; 2 Outcomes and Impact Services, Texas Children’s Hospital, Baylor College of Medicine, Houston, TX USA; 3 Department of Pediatric Orthopaedic Surgery, Texas Children’s Hospital, Baylor College of Medicine, Houston, TX USA

**Keywords:** Developmental dysplasia hip, Spica, MRI, Abduction angle

## Abstract

**Purpose:**

Spica magnectic resonance imaging (MRI) is an established technique for postoperative determination of hip reduction in patients treated for developmental dysplasia of the hip (DDH). A hip abduction angle >55° is considered excessive and has been associated with epiphyseal osteonecrosis. Our purpose was to establish objective criteria for measuring hip abduction angles on MRI after hip reduction and spica casting in patients with DDH, and evaluate reproducibility and reliability of angle measurement using these criteria.

**Methods:**

Forty patients with DDH at our institution who underwent spica MRI after hip reduction between 3 April 2008 and 3 March 2015 were identified. Hip abduction angles were measured on proton density axial images as follows. A transverse line was drawn connecting the posterior ischial tuberosities. A second line was drawn medially along the distal femoral diaphysis, and the angle between these two lines was measured; this value was subtracted from 90°, yielding the degree of abduction from midline. Measurements were independently performed by three faculty radiologists, one orthopedist, and one radiology resident. Inter-reader and intra-reader reliability was assessed using intraclass correlation (ICC), with 0 representing no agreement and 1 representing perfect agreement.

**Results:**

For inter-reader reliability, the ICC of the five physicians was 0.89 (95 % CI 0.84–0.92). For intra-reader reliability, the ICC of the five physicians ranged from 0.90−0.97 (95 % CI 0.85–0.98). The mean standard deviation of hip abduction angle measurement among readers was 3.6°.

**Conclusion:**

The proposed hip abduction angle measurement criteria for spica MRI are both reproducible and easy to perform. The high ICC and low standard deviation of independently evaluated hip abduction angles indicates high reproducibility of measurement. This applies to both inter- and intra-reader reliability.

## Introduction

Developmental dysplasia of the hip (DDH) is a spectrum disorder related to abnormal positioning and development of the femoral head and acetabulum [1]. In a subset of patients who present late or fail treatment with passive motion orthotic devices such as Pavlik harnesses, closed or open reduction with spica casting with the hips in abduction is the preferred method of treatment [2]. The degree of hip abduction from midline is determined clinically in the operating room based on stability of reduction [3]. Although there is some disagreement in the literature as to the ideal amount of abduction, the degree of abduction is generally considered excessive when the angle is >55°–60° [4]. Excessive hip abduction may be associated with a higher incidence of epiphyseal osteonecrosis [5].

Magnetic resonance imaging (MRI) has become the preferred technique to evaluate adequacy of reduction in this setting after hip reduction and spica placement [6, 7]. MRI has several advantages over other imaging modalities. These include a lack of ionizing radiation, superior soft tissue delineation, multi-planar characterization of hip location, and the ability for the study to be performed without sedation. Additionally, with administration of intravenous contrast, MRI can be used to estimate epiphyseal perfusion [8].

Given the serious morbidity related to epiphyseal osteonecrosis, it is important to use an objective method of measuring hip abduction angles in patients who undergo post-reduction MRI, rather than the clinical examination alone. The purpose of this study was to (1) establish definitive, objective criteria for measuring hip abduction angle on MRI after hip reduction and spica casting in patients with DDH, and (2) evaluate the reproducibility and reliability of angle measurement. In order to quantify the latter, we used the intra-class correlation (ICC), a statistical representation of inter-reader and intra-reader reliability.

## Materials and methods

Approval from our Institutional Review Board was obtained for this retrospective study. For this type of study, formal consent is not required. A text word search of the institution’s radiology electronic medical record using the query ‘Spica AND Modality: (MR)’ yielded 40 patients (average age 17.3 months, range 4–64 months; 13:27 male:female) who underwent spica MRI after hip reduction for DDH between 3 April 2008 and 3 March 2015. Five additional patients incorrectly identified based on the above query had never been diagnosed with DDH, did not have a spica MRI examination stored in our PACS systems, or both, and were thus excluded from the study. Many of the 40 patients included in the study underwent multiple spica MRI examinations either due to failed initial reduction and subsequent additional surgical procedure, or for repositioning of the spica cast and subsequent re-imaging to ensure appropriate hip location on the day of the initial procedure. The final MRI on the day of the most recent surgery (if multiple surgeries on different dates) for each patient was analyzed in this study.

The Spica MRI protocol that we use at our institution includes proton density fat-saturated axial and coronal images. Post-contrast coronal and axial images were also obtained if there were no surgical implants, or if the surgeon specifically requested contrast administration. All studies were performed on a 1.5 T MRI platform.

**Fig. 1:**
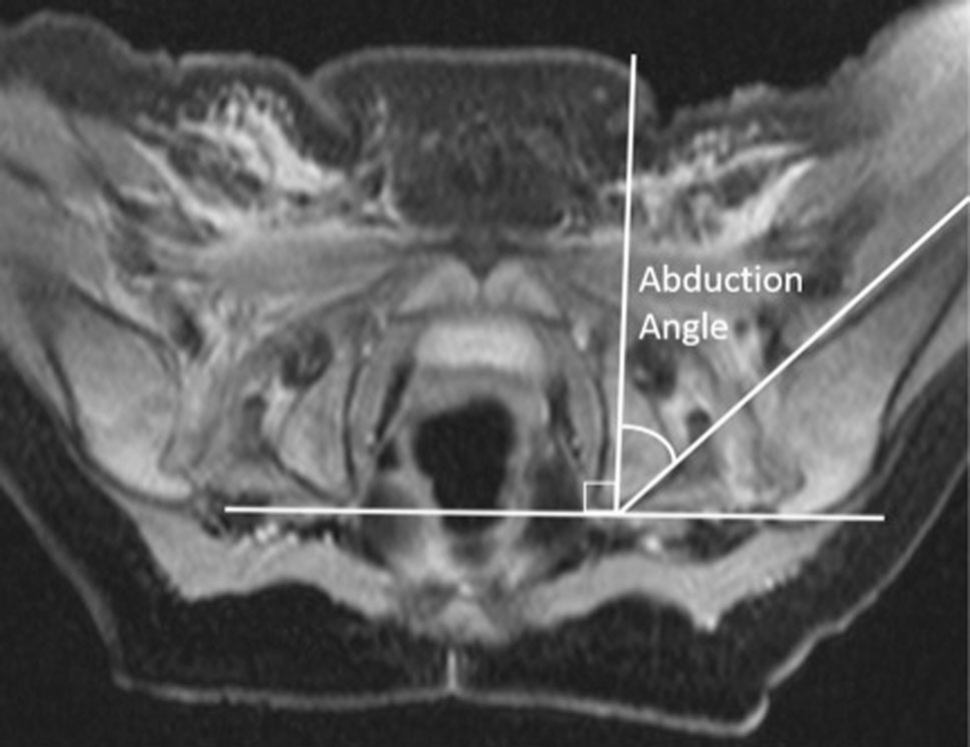
A 5-month-old girl with DDH after spica cast placement. Proton density fat saturated sequence of the pelvis illustrates slice selection, line determination, and technique for measuring hip abduction angle

For each hip, abduction angles were measured on axial images of a proton density MRI sequence as follows: a transverse line was drawn connecting the posterior-most elements of the ischial tuberosities. A second line was drawn anteriorly along the medial aspect of the distal femoral diaphysis, and the angle between these two lines was measured; this value was then subtracted from 90° to determine the degree of abduction from midline (see Fig. 1). To improve homogeneity of hip abduction angle measurements by MRI, all reviewers practiced on three test images before evaluating the cohort images, and had these practice images available for reference when necessary. When evaluating the cohort images, the readers were not given a specific image to measure, and instead had to choose the correct image from a proton density axial MRI sequence that best allowed them to identify the pertinent landmarks in question. The landmarks themselves were not pre-marked in any way; the readers were asked to measure the cohort images just as they did on the practice images.

Abduction angles were independently measured by five readers—three faculty pediatric radiologists, one faculty pediatric orthopedist, and one diagnostic radiology resident (PGY-3). The five measurements for each hip were then averaged to obtain a final measurement for each hip. The measurements of each individual reader were compared to each other as well as to the averaged final measurement to ensure there were no outliers. The agreement of the five readers was assessed via the intra-class correlation, a statistical representation of inter-reader reliability. ICC was calculated using SAS Version 9.3 statistical analysis software. Intra-reader reliability was calculated on a separate occasion >6 months from the initial review. The five readers above independently re-measured hip abduction angles of the same patient cohort; each set of individual reader measurements was then compared to the same reader’s measurements from earlier in order to assess intra-reader reliability, quantified by the ICC.

The ICC is used to assess the consistency, or conformity of measurements made by multiple observers, measuring the same quantity of a continuous variable [9]. A value of 1 represents perfect inter-reader reliability, whereas a value of 0 represents no agreement whatsoever. ICC can be interpreted as follows: 0–0.2 indicates poor agreement; 0.3–0.4 indicates fair agreement; 0.5–0.6 indicates moderate agreement; 0.7–0.8 indicates strong agreement; and >0.8 indicates near-perfect agreement [10].

## Results

Of the 40 patients in our study group, 17 patients (average age 23.6 months, range 8–64 months; 6:11 male:female) underwent open reduction, and 23 patients (average age 12.6 months, range 4–41 months; 7:16 male:female) underwent closed reduction. Eighty individual hips (two for each of the 40 study participants) were evaluated postoperatively after placement in a spica cast using a 1.5 T MRI; 14 hips were excluded due to MRI susceptibility artifacts from surgical implants placed during open reduction, which prevented accurate measurement of the hip abduction angle. Therefore, the final study population included 66 hips.

An ICC value and associated 95 % confidence interval were calculated to assess inter-reader reliability for the following sub-groups of readers, all of which exhibited near-perfect agreement. The four radiologists exhibited the highest ICC of 0.92 (0.89, 0.95). The three faculty radiologists had an ICC of 0.90 (0.86, 0.93). All five readers together had an ICC of 0.89 (0.84, 0.92) (Table 1).

**Table 1:** ICC (intraclass correlation) of the five readers, including reader subgroups. ICC measures inter-reader reliability, the degree of agreement among readers

Readers	ICC (95 % CI)
All five readers	0.89 (0.84, 0.92)
Four radiologists	0.92 (0.89, 0.95)
Three attending radiologists	0.90 (0.86, 0.93)

An ICC value and associated 95 % confidence interval were similarly calculated for each of the five individual readers in order to assess intra-reader reliability. The ICC measurement of intra-reader reliability was high, ranging from 0.90−0.97 (0.85–0.98) among the five readers (Table 2).

**Table 2:** ICC of the five individual readers, measuring intra-reader reliability

Reader	ICC (95 % CI)
Radiologist 1	0.90 (0.85, 0.94)
Radiologist 2	0.92 (0.87, 0.95)
Radiologist 3	0.93 (0.89, 0.95)
Orthopedist	0.92 (0.87, 0.95)
Radiology resident	0.97 (0.95, 0.98)

**Fig. 2:**
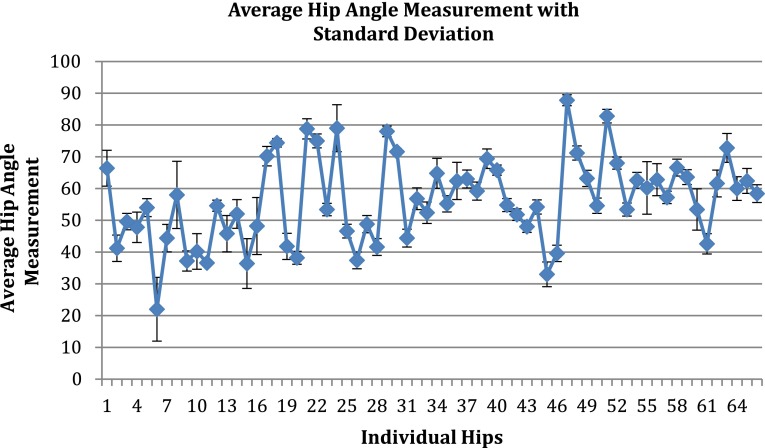
Graph of average hip abduction angle measurements (in degrees), with standard deviation

The average hip abduction angle for the 66 individual hips was 56.2° (range 13°–90°) (Fig. 2) For each of the 66 hips, the standard deviation of the five individual reader measurements relative to the average of the five measurements was calculated (range 0.49°–10.59°). These 66 standard deviations were then averaged, yielding a mean standard deviation of 3.62°.

## Discussion

Our proposed spica MRI abduction angle measurement criteria are straightforward and easy to perform. The high ICC and low standard deviation of independently measured spica MRI hip abduction angles indicate a high inter-reader and intra-reader reliability and reproducibility of measurement between physicians of different specialties and different levels of training. Spica MRI abduction angle measurements may be an objective, reproducible metric tool to identify hips at risk for epiphyseal osteonecrosis based only on excessive abduction.

Our method of measuring abduction angles differs from previously described methods that were created using computed tomography (CT), such as those used by Browning et al. [11] and Stanton et al. [12]. These CT-based methods used the trans-triradiate line as a horizontal landmark. We chose to use the posterior ischium for our measurements because it was an easier landmark to identify by MRI using multiple observers with different levels of training. Our measurements had relatively higher inter-observer correlation compared with published CT data [13]. Because our measurement criteria are easier to reproduce compared with previously published methods, they may be more widely adopted by both pediatric orthopedists and radiologists in their interpretation of spica MRI examinations.

The significant morbidity of epiphyseal osteonecrosis following hip reduction and spica placement, which may be related to excessive hip abduction angle in this setting, necessitates an objective and accurate measurement of the hip abduction angle. An excessive hip abduction angle has long been postulated to compromise blood supply to the femoral epiphysis by either compressing the deep medial circumflex femoral artery or by exerting supra-physiologic pressure on the femoral head related to hip incongruence [14]. MRI has been shown to improve the ability to identify diminished blood flow [15]. Jaramillo et al. investigated how abnormal gadolinium enhancement of the femoral head on MRI after hip reduction and spica placement could detect decreased blood flow and allow ‘timely correction of the angle of abduction to prevent avascular necrosis and proximal femoral abnormalities’ [16].

The general consensus in pediatric and orthopedic literature is that hip abduction angles >55°–60° invoke an increased risk of epiphyseal osteonecrosis. Smith et al. showed a significant risk of subsequent development of avascular necrosis (AVN) when the hip abduction angle on post-reduction CT scan was >55° [17]. In fact, 33 % of hips that were abducted >55° in Smith’s study developed AVN, whereas no hips that were abducted <55° developed AVN. To our knowledge, our study is the first to demonstrate that hip abduction angles by MRI can consistently be acquired with good inter-observer reliability. MRI provides exquisite soft tissue delineation, lacks ionizing radiation, and can identify impediments to successful reduction without the need for sedation. It is a superior modality for assessment of post-reduction hip abduction angle, and is more objective than clinical measurement performed in the operating room.

A reproducible method for determining hip abduction angles after a child undergoes treatment for DDH has important clinical and research implications. First, excessive hip abduction may require surgical revision. In this case, an objective evaluation of hip abduction angles post-revision can easily be performed with our measurement criteria. Second, our straightforward criteria for spica MRI hip abduction angle measurements may be readily adopted for follow-up research studies, which may seek to determine the relationship between excessive hip abduction angles and the risk for development of epiphyseal osteonecrosis in this setting.

This study is limited in its scope in regard to what an optimal post-reduction hip abduction angle should be. This study describes the reliable technique of measuring hip abduction angles. Future studies are required to put these angle measurements in context with long-term complications of reduction and spica casting, including epiphyseal osteonecrosis, long-term concentric reduction of the hip, and early osteoarthritis. An easy to perform, reliable method for hip abduction angle measurement will add additional value to the MRI examination that is going to be performed either way.

## Conclusion

Our study has set forth objective, teachable, and reproducible MRI measurement criteria for evaluation of hip abduction angles after hip reduction and spica casting in patients with developmental dysplasia of the hip. Additionally, the high ICC among and between different readers with different levels of training indicates a high reproducibility of measurement. Our method of determining MRI abduction angles uses modified measurement criteria compared with CT criteria that have been previously published [11]. Given the significant risk of epiphyseal osteonecrosis associated with excessive hip abduction angles in this setting, this angle should be routinely included in all spica MRI interpretations.
